# Comparison of the use of comprehensive point-of-care test panel to conventional laboratory process in emergency department

**DOI:** 10.1186/s12873-018-0198-x

**Published:** 2018-11-19

**Authors:** Meri Kankaanpää, Marika Holma-Eriksson, Sami Kapanen, Merja Heitto, Sari Bergström, Leila Muukkonen, Veli-Pekka Harjola

**Affiliations:** 1 Emergency Medicine, University of Helsinki and Department of Emergency Medicine and Services, Helsinki University Hospital, Haartmaninkatu 4, PL 340, 00029 Helsinki, Finland; 2 Department of Clinical Chemistry and Haematology, Helsinki University Hospital, HUSLAB, Helsinki, Finland

**Keywords:** Laboratory testing, Point-of-care testing, Emergency department, Length of stay, Discharge

## Abstract

**Background:**

In this study, we hypothesized that point of care testing (POCT) would reduce length of stay (LOS) in emergency department (ED) when compared to central laboratory testing and be a factor in patient discharge destination.

**Methods:**

A single centre observational study was performed in ED non-ambulatory patients. Blood testing was performed either with POC instruments for blood gases and chemistry panel, full blood count, and CRP, or at central laboratory, or as a combination of both. Blood draw and POCTs were performed by experienced nurses.

**Results:**

During the 4-week study period, 1759 patients underwent sample testing (POCT: *n* = 160, central lab: *n* = 951; both *n* = 648). Median waiting time for blood sampling was 19 min less in POCT than central laboratory (0:52 (95% confidence interval (CI) 0:46–1:02) vs. 1:11 (95% CI 1:05–1:14), *p* < 0.001). POCT results were available faster in both discharge groups, as expected. When imaging was not required, patients in POCT group were discharged home 55 min faster (4:57 (95% CI 3:59–6:17) vs. 5:52 (95% CI 5:21–6:35), *p* = 0.012) and 1 h 22 min faster when imaging was performed (5:48 (95% CI 5:26–6:18) vs. 7:10 (95% CI 6:47–8:26), *p* = 0.010). Similar reduction in sampling time and LOS was not seen among those admitted to hospital.

**Conclusions:**

POCT shortened the laboratory process and made results available faster than the central lab. This allowed patients to be discharged home quicker. Thus, with proper training and education of the ED care team, POCT can be used as an effective tool for improving patient flow.

## Background

High quality and patient-centered care requires early diagnosis, which is achieved by eliminating unnecessary pre- and post-analytical delays. Blood testing and diagnostic imaging are essential routines of ED, and especially blood testing is associated with prolonged length of stay [1]. The laboratory turn-around time for results from central laboratories (CL) can be over 60 min, compared to 10 to 15 min for point-of-care bedside testing (POCT).

Many studies on POCT, focused on selected tests and limited patient populations, have suggested reduced length of stay (LOS) [2–4]. A recent study by Singer et al. focusing on critical care patient population reported reduced LOS using similar iSTAT POCT equipment as used in this study [5]. Some have also reported that POCT strategy alone has not necessarily improved LOS or that it has had effect on only a certain group of patients [6]. Most studies on POCT focus on diagnostic accuracy [7, 8] instead of process improvement. The full benefit of POCT is acquired when it is implemented together with process redesign [9, 10]. When properly used, POCT can result in a number of benefits in the quality and efficiency of care [11].

Focusing on the process management point of view, we hypothesized that POCT would reduce LOS in emergency department (ED) when compared to central laboratory testing and be a factor in patient discharge destination; home or hospital.

## Methods

### Study design

The study was performed as a prospective single centre study in a Finnish metropolitan hospital with approximately 61,000 annual visits at the hospital’s ED. The patient population consists of specialty care adult patients, and outside office hours also primary care patients. Central laboratory is located at the same site but operated separately, with ED being one of their clients. Laboratory personnel take samples which are then transported via a pneumatic tube system for analysis.

**Fig. 1:**
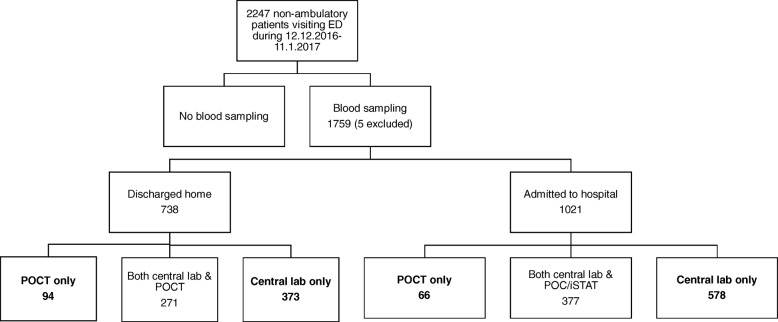
Study patient population. The POCT only groups were compared to the Central lab only group

This study focuses on non-ambulatory patients who needed blood sampling (Fig. 1). Urine, fecal and cerebrospinal fluid testing were excluded from the analysis. Also, only laboratory results ready before discharge were taken into account. The study period was 1 month, lasting from Dec 12, 2016 to Jan 11, 2017.

### Study protocol

The central laboratory personnel validated the POCT instruments, and their diagnostic accuracy was agreed to be at a clinically acceptable level. Nurses were trained to take blood samples and use the POCT equipment. Each nurse went through two training sessions (2 h each), and did a skills demonstration test before qualification. The nurses were advised to take blood samples from patients during initial assessment and management. Physicians were informed about the project. POC testing was instructed to be the primary analysis method for each non-ambulatory patient. Central lab was used either alone, when samples were taken by a biomedical laboratory scientist, or in addition to POCT, when both samples were taken by an ED nurse. Both central lab and ED nurses operated 24 h a day, 7 days a week.

Blood testing was performed either with POC instruments iSTAT (Abbott) for blood gases and chemistry panel, and PocH-100i (Roche) for blood count, and Afinion (Alere) for CRP; or at central laboratory; or combination of both. Three iSTAT testing devices were used for sodium, potassium, creatinine, urea, ionized calcium, blood gas analysis and glucose. Troponin I was excluded from the protocol due to low detection limit. ISTAT devices were given out to three patient areas of 9 to 10 beds having two nurses each. No extra personnel resources were added.

The POCT results were sent electronically to the central laboratory database and reported through the electronic medical record system. Data were collected from hospital, laboratory and imaging databases. The search was limited to patients who visited the ED during the length of the project and whose laboratory and radiology tests were ordered by the ED.

### Outcome measures

Waiting time from admission to laboratory sampling, diagnostic imaging (like x-ray and CT scans) and discharge (LOS) was calculated. Discharge time point was when the patient left the ED, since it was not possible to identify the time the patient was ready for the discharge. One patient may have had multiple laboratory time stamps. The first sampling time per patient visit was identified, and laboratory results were considered ready when all results before discharge were ready. Results were often ready at different times due to multiple analytical laboratory devices used. The central laboratory process registered the actual sampling time, POCT sampling time was defined as the time when analysis started. Due to this, 5 to 10 min should be reduced from POCT admission to sampling time.

### Data analysis

**Fig. 2:**
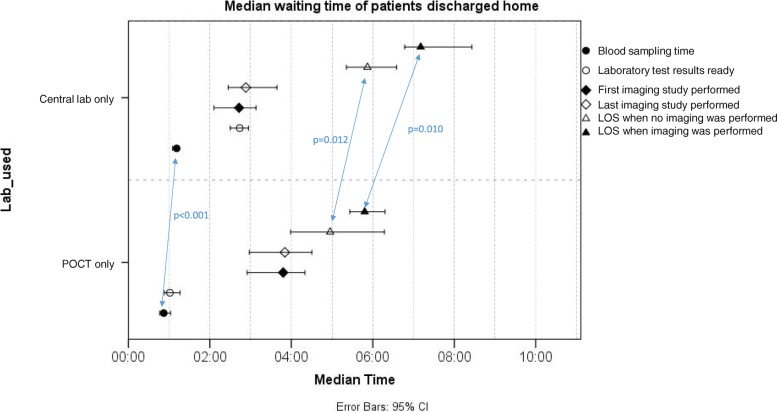
Median waiting times from admission to blood sampling, laboratory results ready, imaging and discharge home with 95% confidence intervals

**Fig. 3:**
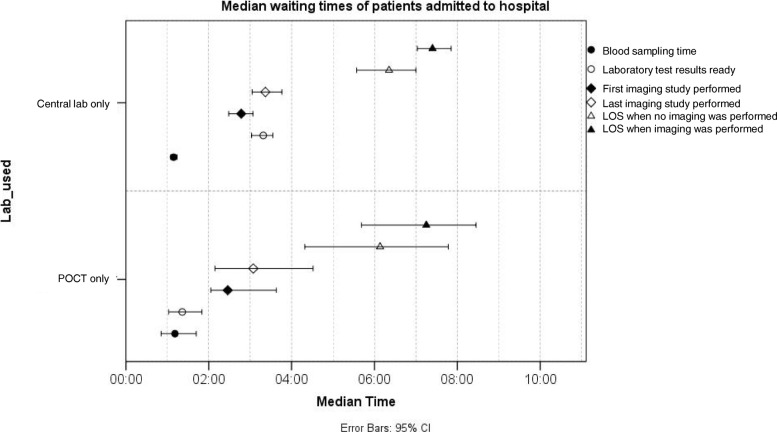
Median waiting times from admission to blood sampling, laboratory results ready, imaging and discharge to hospital with 95% confidence intervals

Median waiting times were calculated since data was positively skewed i.e. had a long right tail. Medians were presented with 95% confidence intervals (95% CI) (Figs. 2 and 3), and 95% CIs were also calculated. Patients discharged home were analyzed separately from patients admitted to hospital. Finally, patient groups including only POC tested patients were compared to those analyzed by central lab only. Patients having both central laboratory testing and POCT were excluded from the study in order to get better comparability between two testing options.

Mann & Whitney U-test was used to test for differences in waiting time distributions between two subgroups, and results were presented as *p*-value. Statistical analyses were performed using SPSS computer software (SPSS Inc., Chicago, IL, USA). All interpretations are based on α = 0.05.

## Results

Age and sex patterns were mainly similar in all subgroups. Patients discharged home were younger than those admitted to hospital ward and had more patients analyzed by POC only in relation to patients admitted to hospital (Table 1).

**Table 1:** Baseline characteristics of study participants

	Discharged home		Admitted to hospital	
	POCT only	Central lab only	POCT only	Central lab only
Number of patients	94	373	66	578
Mean age, years	63	59	68	69
Females %	57%	54%	59%	52%
Diagnosis				
Infection	9 (10%)	88 (24%)	14 (21%)	233 (40%)
Cardiopulmonary	24 (26%)	105 (28%)	12 (18%)	104 (18%)
Neurology	16 (17%)	60 (16%)	10 (15%)	50 (9%)
Other	10 (11%)	52 (14%)	7 (11%)	65 (11%)
Abdominal	17 (18%)	28 (8%)	6 (9%)	53 (9%)
Musculoskeletal	6 (6%)	21 (6%)	9 (14%)	32 (6%)
Unspecific complaint	6 (6%)	9 (2%)	6 (9%)	26 (4%)
Alcohol	6 (6%)	10 (3%)	2 (3%)	15 (3%)

Compared to patients discharged home from ED, the ones admitted to hospital more often had infectious diseases and less often cardiopulmonary disease. The patients who required central laboratory testing had more often infectious diseases and less often abdominal complaints than the patients in POC group (Table 1) The group of patients having both POC and central laboratory testing (excluded from study) had a diagnosis distribution relatively similar to the group tested by central laboratory only. Approximately 70% of non-ambulatory patients went through radiological imaging during their stay in ED.

Figure 2 shows waiting times of patients discharged home. Median waiting time for blood sampling was 19 min less for POC patients when sampling was done by ED nurses, compared to central laboratory patients (0:52 (95% CI 0:46–1:02) vs. 1:11 (95% CI 1:05–1:14), *p* < 0.001). In actuality, the difference is even larger, since POC blood samples were taken 5 to 10 min before the first time stamp. Similar reduction in sampling waiting time was not seen in the patient group admitted to hospital (Fig. 3).

POCT results were available significantly faster in both discharge groups, as expected. POCT results were completed 1 h 1 min faster in the discharged home group (00:06 (95% CI 0:05–0:07) vs. 1:07 (95% CI 1:01–1:13), *p* < 0.001) and 1 h 39 min faster in the admitted to hospital group (0:06 (95% CI 0:04–0:07) vs. 1:45 (95% CI 1:33–1:57), *p* < 0.001). One hundred and-forty POCT patients from total 160 had results ready in less than 15 min. The 20 patients above 15 min had had multiple POC tests taken, which were completed at different times.

However, it was only the patients discharged home (Fig. 2), who were discharged 55 min faster without imaging (4:57 (95% CI 3:59–6:17) vs. 5:52 (95% CI 5:21–6:35), *p* = 0.012) and 1 h and 22 min faster with imaging (5:48 (95% CI 5:26–6:18) vs. 7:10 (95% CI 6:47–8:26), *p* = 0.010). Similar reduction in LOS was not seen among those admitted to hospital (Fig. 3).

## Discussion

In our study, only those discharged home were fully able to benefit from POCT and were discharged 55 min (no imaging) or 1 h 22 min (imaging) earlier. The time from ED admission to sampling and results ready to discharge will not be reduced without changes in working practice. Patients’ admittance to hospital/care unit may be delayed due to need for additional diagnosis, or lack of availability of hospital beds.

Analysis did not reveal any specific group with whom POCT cannot be used. The POCT group represented all diagnostic subgroups and the group having both POC and central lab testing was similar to those tested only by central lab.

The study results are in agreement with previous studies evaluating POCT impact on waiting times and LOS. Singer et al. evaluated POCT impact on critical care patients using iSTAT devices (Hb, hematocrit, TnI, lactate, BNP, INR). They reported a 33-min reduction in median LOS in patients admitted during office hours (not statistically significant) and an 87-min reduction in median LOS in patients presenting at all times who required a CT with IV contrast [5]. Jang et al. [12] compared ED length of stay when noncritical nonpediatric patients were assigned to a comprehensive point-of-care test to those with central laboratory testing. They reported a 22 min shorter median LOS compared to CL group (with a LOS approx. 6 h) [12]. In our previous study with ambulatory patients, introduction of POCT reduced median LOS by 29 min, and the Early Assessment Team (EAT) model reduced median LOS further by 17 min. EAT consists of a consultant emergency physician and a nurse, aiming at define the need for laboratory testing and imaging fast in order to make process faster and safe for the patient. Altogether, the process was expedited by 46 min compared to original setup [9]. Lee-Lewandrowski et al. [13] evaluated LOS before and after implementation of POCT laboratory testing glucose, urine dipstick, hCG, and cardiac markers. They reported a 41-min decrease in patient LOS. Singer et al. [14] reported a 64-min reduction in LOS in a similar study [13].

Thus, with proper training and education of the ED care team, POCT can be used as an effective tool for managing patient flow in ED. However, best value for investment is received when process efficiency is also optimized. POCT together with efficient triage, senior consultant support and best care pathways would probably decrease LOS further.

### Limitations

Patients were passively divided into each study group and not actively randomized. This may affect the comparability of groups, and it also resulted in relatively big differences in group sizes.

Influenza epidemic landed during project time, which challenged ED resources and may have had an impact on patient population characteristics. The number of inpatient beds is limited, and this affects the admitted to hospital group LOS.

## Conclusions

The results of this study demonstrate that POCT provides laboratory results faster than the traditional central laboratory process. However, process improvement is needed in order to take advantage of this faster availability of laboratory results.

In this population, POCT shortened the length of stay in ED in patients discharged home. Further studies are needed to show whether achieving laboratory results earlier translates to improved patient safety.

## Abbreviations

ED: Emergency department
LOS: Length of stay
POCT: Point-of-care testing
